# Glycaemic thresholds for counterregulatory hormone and symptom responses to hypoglycaemia in people with and without type 1 diabetes: a systematic review

**DOI:** 10.1007/s00125-022-05749-8

**Published:** 2022-07-22

**Authors:** Clementine E. M. Verhulst, Therese W. Fabricius, Steven Teerenstra, Peter L. Kristensen, Cees J. Tack, Rory J. McCrimmon, Simon Heller, Mark L. Evans, Stephanie A. Amiel, Ulrik Pedersen-Bjergaard, Bastiaan E. de Galan, on behalf of the Hypo-RESOLVE consortium

**Affiliations:** 1 Department of Internal Medicine, Radboud University Medical Centre, Nijmegen, the Netherlands; 2 Department of Endocrinology and Nephrology, Nordsjællands Hospital, Hillerød, Denmark; 3 Section Biostatistics, Department for Health Evidence, Radboud Institute for Health Sciences, Radboud University Medical Centre, Nijmegen, the Netherlands; 4 Department of Clinical Medicine, Faculty of Health and Medical Sciences, University of Copenhagen, Copenhagen, Denmark; 5 School of Medicine, University of Dundee, Dundee, Scotland; 6 Department of Oncology and Metabolism, University of Sheffield, Sheffield, UK; 7 Wellcome Trust/MRC Institute of Metabolic Science, University of Cambridge, Cambridge, UK; 8 Department of Diabetes, School of Life Course Sciences, Faculty of Life Sciences & Medicine, King’s College London, London, UK; 9 Department of Internal Medicine, Division of Endocrinology, Maastricht University Medical Centre, Maastricht, the Netherlands; 10 CARIM School for Cardiovascular Diseases, Maastricht University, Maastricht, the Netherlands

**Keywords:** Counterregulatory hormones, Diabetes, Glycaemic thresholds, Human, Hyperinsulinaemic–hypoglycaemic stepped clamp, Hypoglycaemia, Symptomatic responses; Systematic review

## Abstract

**Aim/hypothesis:**

The physiological counterregulatory response to hypoglycaemia is reported to be organised hierarchically, with hormone responses usually preceding symptomatic awareness and autonomic responses preceding neuroglycopenic responses. To compare thresholds for activation of these responses more accurately between people with or without type 1 diabetes, we performed a systematic review on stepped hyperinsulinaemic–hypoglycaemic glucose clamps.

**Methods:**

A literature search in PubMed and EMBASE was conducted. We included articles published between 1980 and 2018 involving hyperinsulinaemic stepped hypoglycaemic glucose clamps among people with or without type 1 diabetes. Key exclusion criteria were as follows: data were previously published; other patient population; a clamp not the primary intervention; and an inadequate clamp description. Glycaemic thresholds for counterregulatory hormone and/or symptom responses to hypoglycaemia were estimated and compared using generalised logrank test for interval-censored data, where the intervals were either extracted directly or calculated from the data provided by the study. A glycaemic threshold was defined as the glucose level at which the response exceeded the 95% CI of the mean baseline measurement or euglycaemic control clamp. Because of the use of interval-censored data, we described thresholds using median and IQR.

**Results:**

A total of 63 articles were included, whereof 37 papers included participants with type 1 diabetes (*n*=559; 67.4% male sex, aged 32.7±10.2 years, BMI 23.8±1.4 kg/m^2^) and 51 papers included participants without diabetes (*n*=733; 72.4% male sex, aged 31.1±9.2 years, BMI 23.6±1.1 kg/m^2^). Compared with non-diabetic control individuals, in people with type 1 diabetes, the median (IQR) glycaemic thresholds for adrenaline (3.8 [3.2–4.2] vs 3.4 [2.8–3.9 mmol/l]), noradrenaline (3.2 [3.2–3.7] vs 3.0 [2.8–3.1] mmol/l), cortisol (3.5 [3.2–4.2]) vs 2.8 [2.8–3.4] mmol/l) and growth hormone (3.8 [3.3–3.8] vs. 3.2 [3.0–3.3] mmol/l) all occurred at lower glucose levels in people with diabetes than in those without diabetes (all *p*≤0.01). Similarly, although both autonomic (median [IQR] 3.4 [3.4–3.4] vs 3.0 [2.8–3.4] mmol/l) and neuroglycopenic (median [IQR] 3.4 [2.8–N/A] vs 3.0 [3.0–3.1] mmol/l) symptom responses were elicited at lower glucose levels in people with type 1 diabetes, the thresholds for autonomic and neuroglycopenic symptoms did not differ for each individual subgroup.

**Conclusions/interpretation:**

People with type 1 diabetes have glycaemic thresholds for counterregulatory hormone and symptom responses at lower glucose levels than people without diabetes. Autonomic and neuroglycopenic symptoms responses are generated at about similar levels of hypoglycaemia. There was a considerable variation in the methodology of the articles and the high insulin doses in most of the clamps may affect the counterregulatory responses.

**Funding:**

This article has received funding from the Innovative Medicines Initiative 2 Joint Undertaking (JU) under grant agreement no. 777460.

**Registration:**

This systematic review is registered in PROSPERO (CRD42019120083).

**Graphical abstract:**

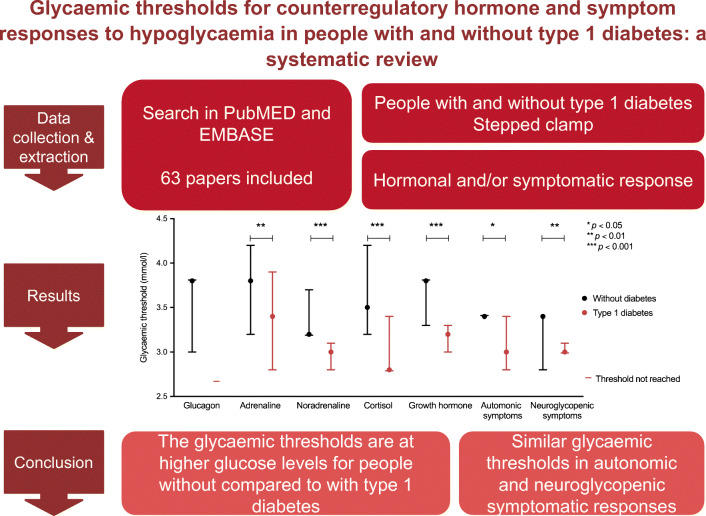

**Supplementary Information:**

The online version contains peer-reviewed but unedited supplementary material available at 10.1007/s00125-022-05749-8.

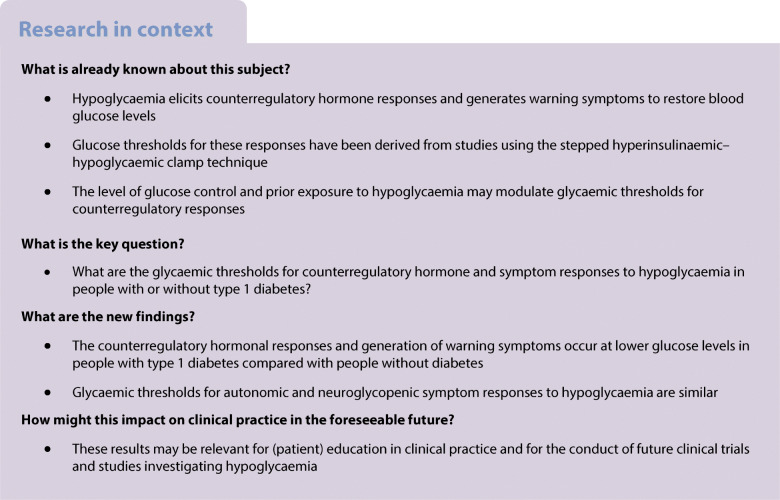



## Introduction

Iatrogenic hypoglycaemia is a continuous threat for most people with type 1 diabetes, occurring weekly or even daily as a consequence of treatment with insulin [1]. Falling glucose levels in the hypoglycaemic range elicit a hierarchically organised counterregulatory response, starting with the suppression of insulin production by beta cells, followed by the release of hormones (glucagon, adrenaline, noradrenaline, cortisol and growth hormone) and finally the appearance of (warning) symptoms to induce a behavioural response (i.e. ingest carbohydrates), aimed at restoring glucose levels. Overall, it is generally considered that hypoglycaemia first elicits autonomic symptoms, hence termed warning symptoms, followed by neuroglycopenic symptoms, usually thought to reflect cerebral glucopenia [2].

The hyperinsulinaemic clamp is a methodology that has been widely employed to study the impact of varying degrees of hypoglycaemia in counterregulatory responses and used to determine the threshold for activation of the individual components [3, 4]. With this method, insulin is infused intravenously at a constant rate sufficient to cause plasma glucose levels to fall, alongside a variable intravenous glucose infusion titrated against frequent glucose measurements to achieve hypoglycaemia at predefined glucose plateaus. During each plateau, counterregulatory hormone concentrations and symptom scores are measured to define the glucose level at which a response can be first detected.

Although reported to be organised hierarchically, the glycaemic thresholds at which these responses occur are not fixed, showing inter- and intra-individual variability, and are influenced by prior exposure to hypo- and hyperglycaemia, as well as by age and duration of diabetes [5, 6]. In addition, factors such as the inability to dissipate insulin, blunted glucagon secretion and reduced catecholaminergic responses in people with type 1 diabetes [7] can influence each component of the counterregulatory hormone and symptom response and so thresholds may differ between those with and without type 1 diabetes. Whether in type 1 diabetes per se, glycaemic thresholds differ from those in people without diabetes remains to be determined. To explore the glycaemic thresholds for counterregulatory hormone and symptom responses, we performed a systematic review on hyperinsulinaemic stepped hypoglycaemic glucose clamps reporting counterregulatory and/or symptom responses in people with or without type 1 diabetes.

## Methods

The search string and selection of publications for this systematic review have been published previously [8]. See electronic supplementary material (ESM Methods) for the search strategy. The protocol is published in PROSPERO (CRD42019120083): https://www.crd.york.ac.uk/prospero/display_record.php?RecordID=120083. All articles with stepped clamps from the systematic review were included in this article. Only English-language articles were included.

### Data sources and search strategy

A literature search was conducted in PubMed and EMBASE in November 2018. All articles available online in the databases and published from 1980 to 2018 were included. The search used a combination of free text words, MeSH (PubMed), and Emtree (EMBASE) terms. All titles and abstracts identified from the electronic search via PubMed and EMBASE were imported to COVIDENCE [8] software, version 1.0, which streamlines the review process. The search strategy (ESM Methods) was developed in collaboration with an information specialist at Nordsjællands Hospital, Denmark, with input from clinicians and academics in the review team. Details on study selection have been published previously [4]. To obtain additional data missing in the articles, we contacted the corresponding authors.

### Data extraction

A total of 3887 articles were identified, 547 full-text articles were assessed for eligibility and 383 were included in the systematic review [4]. Out of these 383 articles, 108 reported a stepped hyperinsulinaemic–hypoglycaemic glucose clamp in people with type 1 diabetes and/or people without diabetes. People were classified as having a reduced awareness of hypoglycaemia when it was stated by the paper or when they had a history of severe hypoglycaemic events. Articles were included if they provided data about glycaemic thresholds or when it was possible to calculate these. Forty-five articles were excluded due to lack of information, resulting in 63 articles being included in the systematic review (ESM Table 1) [3, 6, 9–69]. Of these, the glycaemic thresholds were provided in 47 papers, and 17 contained sufficient data (mean±SD of baseline and every step) to calculate these for at least one counterregulatory hormone (i.e. glucagon, adrenaline, noradrenaline, cortisol and/or growth hormone) or for symptom responses. In one of the included articles, the glycaemic threshold was provided for some of the counterregulatory hormones and calculated for others. Of the 47 articles that provided the glycaemic thresholds, 42 articles determined the thresholds based on the 95% CI, and three articles used ANOVA. In two articles, information on how glycaemic thresholds were determined was missing. Of the 42 papers that based the glucose thresholds on 95% CI, 17 articles did this on the basis of exceeding the 95% CI of data derived from euglycaemic control experiments and 23 papers of data derived from baseline measurements. Two additional papers used a euglycaemic control clamp and although those two papers did not mentioned whether the data were derived from the control clamp or from baseline measurements, we assumed that the data derived from the control clamp. We used the glucose values of the clamped phases below and above the provided or calculated glycaemic threshold for the survival analysis (see Statistics). For example, when a paper used the glucose steps 4.0, 3.0 and 2.5 mmol/l and found the threshold for adrenaline to be at a glucose level of 3.5 mmol/l, the glucose steps 4.0 and 3.0 mmol/l were included in the survival analysis. For people with type 1 diabetes, thresholds for glucagon responses were excluded because a response was often absent or not provided (*n*=58). Symptom scores were classified as autonomic (sweating, anxiety, tremor, palpitations, feeling hot and tingling) or neuroglycopenic (difficulty speaking, confusion, dizziness, irritability, blurred vision and drowsiness). See the Preferred Reporting Items for Systematic Review and Meta-Analyses (PRISMA) flow diagram for an overview of the process (ESM Fig. 1). Quality assessment was ensured by two reviewers extracting information, and by crosschecking of all the included articles. In studies where glucose measurements were derived from whole blood, these were converted to plasma glucose values, assuming plasma glucose levels to be 11.1% higher than whole blood measurements [70].

### Statistics

Results are shown with standard descriptive statistical methods. Continuous data are shown as means with SD, which for readability reasons applies to both normal and non-normal distributed data. We used data provided by the articles on both the glucose value below and the glucose value above the determined glycaemic thresholds (i.e. interval-censored data). When these were not provided by the article, we calculated these values from the plasma glucose level at which the counterregulatory hormone or symptom response first exceeded the 95% CI of the mean baseline measurement. When needed, we determined the 95% CI from the provided sample size. As the thresholds are not known by their value, but only up to the interval in which they are censored, statistical methods such as *t* tests and linear regression are not applicable. Methods to deal with interval-censored data have been developed in a survival data context and use survival data terminology [71]. Therefore, we described thresholds using median and IQR, and used logrank tests to compare the thresholds in people with or without diabetes and people with normal awareness of hypoglycaemia or impaired awareness of hypoglycaemia. We assessed a potential effect of HbA_1c_ and diabetes duration on glucose thresholds by including these variables as explanatory variables in Cox regression analyses for interval-censored data. Note that we apply these interval-censored survival data techniques such that the ‘event’ is the occurrence of a threshold and, instead of ‘follow-up’ time that increases until the ‘event’ has happened we have the glucose level increasing until a threshold has occurred. A level of statistical significance was set to 5% (two-sided). Statistical analyses were performed using IBM SPSS Statistics for Windows, version 25 (IBM Corp., Armonk, NY, USA) and SAS 9.4 (SAS Institute, Cary, NC, USA).

## Results

The 63 included studies involved 1332 participants with a median of 16 participants (range 6–90) per study, of which 11 exclusively enrolled people with type 1 diabetes, 26 only participants without diabetes, and 26 included both. The participants were generally young, had a normal BMI, and were more often male than female (Table 1).

**Table 1:** Baseline characteristics of the participants

Characteristic	Studies on people without diabetes (*n*=51)	Studies on people with type 1 diabetes (*n*=37)
No. of participants	733	599
Age, years	31.1±9.2	32.7±10.2
Male sex, *n* (%)	531 (72.4)	404 (67.4)
HbA_1c_		
mmol/mol	33.3±0.3	70.5±20
%	5.2±0.3	8.6±1.9
SD^a^	1.0±1.3	1.5±1.0
Diabetes duration, years	-	14.6 ± 6.5
BMI, kg/m^2^	23.6±1.1	23.8±1.4

Data are shown as *n* (%) or mean±SD

^a^Average SD as reported across studies

The hypoglycaemic clamps were designed with a variable number of steps, with the most frequently used number of steps after the normoglycaemic phase being four (*n*=27, 43%) and three (*n*=18, 29%). In the four-step clamps, the mean achieved plasma glucose levels were 4.4±0.1 mmol/l, 3.7±0.1 mmol/l, 3.1±0.1 mmol/l and 2.5±0.1 mmol/l, respectively. For the three-step clamps, the mean achieved plasma glucose levels were 4.6±0.4 mmol/l, 3.7±0.1 mmol/l and 3.0±0.1 mmol/l. Twenty-six articles used whole blood to measure blood glucose values and 37 measured glucose values in plasma.

### Glycaemic thresholds for counterregulatory hormone responses

**Fig. 1:**
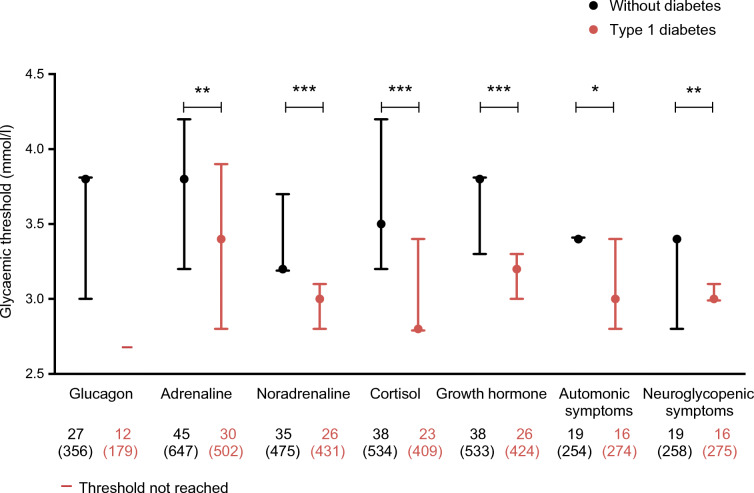
Median glycaemic thresholds for counterregulatory hormone release and symptom responses to hypoglycaemia in people with or without type 1 diabetes. Data are presented as median with IQR. The numbers below the *x*-axis indicate the number of studies (participants). *p<0.05, **p<0.01, ***p<0.001

**Fig. 2:**
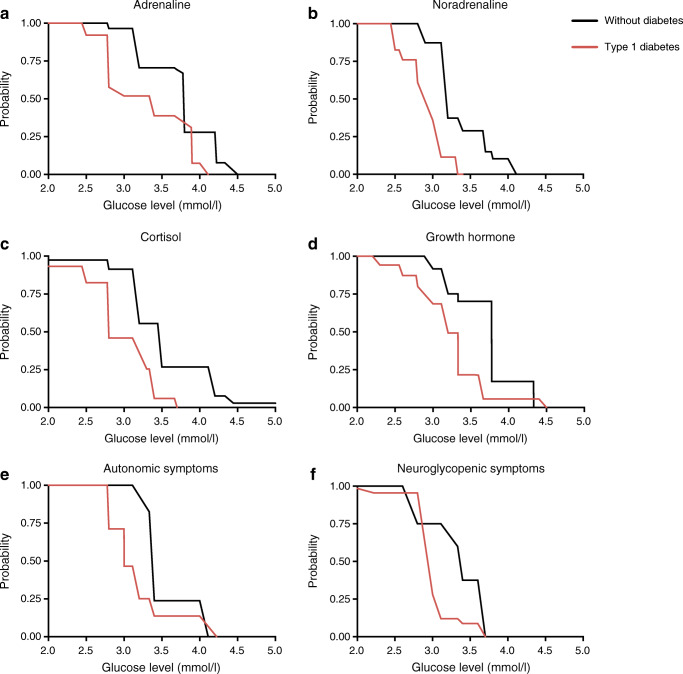
The glycaemic thresholds for the release of the counterregulatory hormones adrenaline (**a**; *p*=0.007), noradrenaline (**b**; *p*<0.001), cortisol (**c**; *p*<0.001) and growth hormone (**d**; *p*<0.001), and for eliciting autonomic (**e**; *p*=0.01) and neuroglycopenic (**f**; *p*=0.007) symptomatic responses are shown in non-parametric complementary cumulative distribution curves (‘survival’ curves). The *p* values refer to the comparison of curves of people with type 1 diabetes and without diabetes with a generalised logrank test for interval-censored survival curves. The values on the vertical axis show the probability that the threshold is larger than the corresponding value on the glucose level axis (horizontal axis)

All but one [6] of the studies (98%) provided data to either calculate or extract the glycaemic threshold for counterregulatory hormone responses to hypoglycaemia. The median (IQR) glycaemic threshold for the glucagon response in people without diabetes was 3.8 (3.0–3.8) mmol/l. The glycaemic thresholds for eliciting hormone responses all occurred at lower glucose levels in people with diabetes than in those without diabetes (all *p*<0.01, Figs 1, 2): 3.8 (3.2–4.2) vs 3.4 (2.8–3.9) mmol/l for adrenaline; 3.2 (3.2–3.7) vs 3.0 (2.8–3.1) mmol/l for noradrenaline; 3.5 (3.2–4.2) vs 2.8 (2.8–3.4) mmol/l for cortisol and 3.8 (3.3–3.8) vs 3.2 (3.0–3.3) mmol/l for growth hormone. This was also true when the analysis was restricted to the studies that examined both people with type 1 diabetes and healthy control individuals (ESM Fig. 2). There were no differences between studies using vs not using euglycaemic control experiments (ESM Fig. 3). Neither the duration of diabetes nor the level of glycaemic control (as measured by HbA_1c_) was associated with the glycaemic threshold level for any of the measured hormones (ESM Table 2).

### Glycaemic thresholds for symptom responses

A total of 25 studies (40%) provided data to either calculate or extract the glycaemic threshold for symptom responses to hypoglycaemia [6, 13, 16–18, 20, 23, 24, 26, 29, 31, 33–37, 46, 51–53, 56, 57, 58, 63, 68]. The median (IQR) glycaemic threshold for the appearance of autonomic symptoms was 3.4 (3.4–3.4) mmol/l in those without diabetes and 3.0 (2.8–3.4) mmol/l in people with type 1 diabetes (*p*=0.01). Similarly, the glycaemic thresholds for the appearance of neuroglycopenic symptoms averaged 3.4 (2.8–N/A) mmol/l and 3.0 (3.0–3.1) mmol/l in people without and with type 1 diabetes, respectively *(p*=0.007) (Figs 1, 2). There were no differences between studies using or not using euglycaemic control experiments, except for a small but significant difference in the neuroglycopenic symptoms in people with diabetes, where the curves of the euglycaemic control experiments were at a lower glucose levels (*p*=0.003; ESM Fig. 3). Glycaemic thresholds did not differ between autonomic and neuroglycopenic symptoms, either in people with diabetes or in those without diabetes. Restricting the analysis to studies that included both people with type 1 diabetes and healthy control individuals or to studies without cognitive function tests (which could potentially alert participant awareness for neuroglycopenic symptoms) did not materially change this. Neither the duration of diabetes nor the level of glycaemic control (as measured by HbA_1c_) was associated with the glycaemic threshold level for symptom responses in people with diabetes (ESM Table 2).

### Sensitivity analyses of awareness of hypoglycaemia status

Based on 13 of the articles that included people with impaired awareness status, the curves in the graph for glycaemic thresholds were mostly at lower glucose levels in people with impaired hypoglycaemic awareness (vs those without): 3.0 (3.0–3.0 mmol/l) vs 3.5 (2.8–3.9 mmol/l) for adrenaline; 2.6 (2.6–N/A mmol/l) vs 3.1 (2.8–3.1 mmol/l) for noradrenaline; and N/A (2.6–N/A) vs 3.3 (3.2–3.7 mmol/l) for growth hormone. This difference did not reach statistical significance for cortisol (2.8 [2.5–3.0 mmol/l] vs 3.3 [2.8–3.4 mmol/l]) (ESM Fig. 4). In addition, for autonomic and neuroglycopenic symptom generation, glucose levels were lower in people with impaired awareness; this difference was only significant for autonomic symptoms (N/A [N/A–N/A mmol/l] vs 3.3 [3.3–3.4 mmol/l], *p*<0.001) and not for neuroglycopenic symptoms (3.0 [3.0–N/A mmol/l] vs 3.0 [3.0–N/A] mmol/l, *p*=0.448) (ESM Fig. 4).

### Sensitivity analyses of glycaemic threshold in three- and four-step clamps

When we restricted the analyses to either the three-step or four-step clamps alone, the differences in glycaemic thresholds between people with vs without diabetes for adrenaline, noradrenaline, cortisol and growth hormone remained largely unchanged (Table 2). The same was true for the glycaemic thresholds for autonomic and neuroglycopenic symptom perception (Table 2). Additionally, when comparing the glycaemic thresholds for counterregulatory responses in three- and four-step clamps separately for each subgroup (participants with type 1 diabetes and healthy participants), there were numerical but no statistically significant differences (Table 2).

**Table 2:** Comparisons of glycaemic thresholds (measured in mmol/l) for counterregulatory hormone or symptom responses to hypoglycaemia in three- and four-step clamps in people with or without type 1 diabetes

Hormone or symptom	Studies on people without diabetes			Studies on people with type 1 diabetes		
	Three-step clamp	Four-step clamp	*p* value	Three-step clamp	Four-step clamp	*p* value
Glucagon	(*n*=6) 3.9 (3.2–3.9)	(*n*=19) 3.8 (3.1–N/A)	0.555	N/A	N/A	N/A
Adrenaline	(*n*=10) 3.9 (3.5–4.0)	(*n*=20) 3.8 (3.3–N/A)	0.415	(*n*=6) 3.2 (N/A)	(*n*=10) 3.5 (3.0–4.0)	0.411
Noradrenaline	(*n*=10) 3.3 (3.3–N/A)	(*n*=15) 3.4 (3.2–3.4)	0.523	(*n*=5) 3.2 (3.0–N/A)	(*n*=9) 3.0 (2.8–3.0)	0.240
Cortisol	(*n*=8) 3.8 (3.2–4.9)	(*n*=25) 3.4 (3.3–4.1)	0.115	(*n*=2) N/A	(*n*=15) 2.8 (2.8–3.3)	N/A
Growth hormone	(*n*=10) 3.9 (3.3–N/A)	(*n*=23) 3.8 (3.4–3.8)	0.351	(*n*=4) 3.4 (2.6–N/A)	(*n*=15) 3.2 (3.0–3.3)	0.724
Autonomic symptoms	(*n*=0) N/A	(*n*=15) 3.4 (3.4–N/A)	N/A	(*n*=2) N/A	(*n*=13) 3.0 (2.8–3.3)	N/A
Neuroglycopenic symptoms	(*n*=7) (3.0–N/A)	(*n*=7) 3.4 (3.4–N/A)	0.196	(*n*=7) 3.0 (3.0–3.0)	(*n*=6) 3.1 (3.0–3.1)	0.554

Data are shown as median (IQR)

*n*=no. of studies included; N/A denotes incalculable due to there being too few data to provide a median (IQR)

## Discussion

This systematic review shows that in people without diabetes, the glycaemic thresholds for release of counterregulatory hormone responses ranged between 3.2 and 3.8 mmol/l, with upper limits of the IQR as high as 4.2 mmol/l, whereas lower glucose levels were required to elicit symptoms of hypoglycaemia. Compared with non-diabetic control individuals, the release of counterregulatory hormones and generation of symptoms occurred at lower glucose levels in people with type 1 diabetes, in part dependent on awareness status but independent of HbA_1c_ and duration of diabetes. In both the subgroup with and the subgroup without type 1 diabetes, glycaemic thresholds for the emergence of autonomic or neuroglycopenic symptoms were about similar within each subgroup.

Based on hypoglycaemic glucose clamp studies dating back to the 1980s, it is commonly assumed that the physiological response to hypoglycaemia in healthy people occurs at glucose levels below 3.9 mmol/l, with the release of glucagon and adrenaline, whereas glucose levels below 3.3–3.5 mmol/l are reported to trigger symptomatic awareness [72]. The current analysis, which collectively combines data from a large number of individual clamp studies involving more than 1300 individuals, shows that on average much lower glucose levels are required to elicit an acute counterregulatory hormone response and symptom response in both people with type 1 diabetes and in those without diabetes (except for the adrenaline response in people without diabetes). As for glycaemic thresholds for autonomic and neuroglycopenic symptoms in people without diabetes, these appear to be elicited at glucose levels of 3.0–3.4 mmol/l and 3.1–3.7 mmol/l, respectively.

In people with type 1 diabetes, we observed glycaemic thresholds at 0.2–0.7 and 0.4 mmol/l lower glucose levels for counterregulatory hormone and symptom responses, respectively, compared with people without diabetes, although with a wide range, suggesting greater variability in those with type 1 diabetes. This large variability in glycaemic thresholds in type 1 diabetes can probably be explained by different prior hypoglycaemia exposure rates between individuals [7], the loss of glucagon secretion within years after diagnosis [9, 10], blunted catecholamine responses and impaired hypoglycaemic awareness [11, 12]. Indeed, we found clear suggestions of glycaemic thresholds for release of particularly adrenaline and appearance of autonomic symptoms to occur at lower glucose levels in people with impaired awareness of hypoglycaemia. These data are in line with studies showing that prior exposure to recurrent hypoglycaemia, which often is the rule in people with impaired awareness, shifts the glycaemic thresholds for counterregulatory responses to lower glucose levels [6, 15].

We found no evidence that strict glycaemic control had an impact on glycaemic thresholds. This is important, since strict glycaemic control, as reflected by low HbA_1c_, is sometimes viewed as a proxy for greater exposure to hypoglycaemia and higher HbA_1c_ as the opposite. Our data underscore, however, that merely directing people with type 1 diabetes to allow their HbA_1c_ to increase is not expected to alter glycaemic thresholds or the associated burden of hypoglycaemia unless the rate of hypoglycaemia drops considerably [6, 32, 60].

Remarkably, we found no evidence for different glycaemic thresholds for the appearance of autonomic or neuroglycopenic symptoms, neither in people with type 1 diabetes nor in those without. These results contrast with the commonly held assumption that the appearance of hypoglycaemic symptoms is hierarchically organised [13, 14]. One explanation for this apparent discrepancy may be the more immediate recognition of autonomic (e.g. sweating and palpitations) as compared with neuroglycopenic (e.g. difficulty in thinking or speaking) symptoms, unless engaged in something requiring information processing, so that the first is usually mentioned as initial symptoms. Additionally, focusing on autonomic symptoms when educating people with diabetes about hypoglycaemia may reinforce such assumptions whereas in fact such a hierarchy in symptomatology seemingly does not exist.

We could not investigate the glycaemic threshold for the glucagon response in people with diabetes. Most studies did not report on glycaemic thresholds for this response to hypoglycaemia in participants with type 1 diabetes, whereas those that did described a large variety in thresholds, including absent responses. In addition, it is possible that some studies did not reach glucose levels that were sufficiently low to elicit a glucagon response. Although the glucagon response is probably retained in adults with a short duration of type 1 diabetes, contributing to their often good glycaemic control combined with low hypoglycaemia risk [15, 16], it is almost universally lost within the first few years after diagnosis [14, 15]. This observation has been linked to progressive loss of pancreatic beta cells, which are thought to control alpha cell responses to hypoglycaemia [73]. Additionally, reliable measurement of glucagon was problematic at the time when most of the studies were performed. However, this should not distract from the fact that in human physiology, the glucagon response plays a prominent role in glucose counterregulation.

In this systematic review, the calculation of glucose thresholds depended on the predefined glucose steps of the studies that were included in the survival curve analysis for interval-censored data. We included both the glucose level of the first counterregulatory response and the level prior to it to obtain a more realistic ‘glycaemic threshold value’. However, it could still be argued whether a stepped clamp is the optimal study design to determine glycaemic thresholds for counterregulatory responses to hypoglycaemia or whether gradually lowering glucose levels would be more precise for defining these cut-off values. Nevertheless, two studies dating back to the 1990s that used such a gradual glucose-lowering method to assess glycaemic thresholds in children with type 1 diabetes reported results that were in line with our findings [17, 18].

Age and diabetes duration did not influence the glycaemic thresholds, which contrasts with previous studies that showed that older age and longer diabetes duration were associated with altered counterregulatory hormone and symptom responses [19–21]. There are two explanations for this apparent discrepancy. First, the mentioned loss of the glucagon response, usually occurring within 5 years after diabetes diagnosis, and the resultant greater exposure to hypoglycaemia may lead to a ‘sudden’ shift of glycaemic thresholds for counterregulatory responses to lower glucose levels [74]. Indeed, those with retained beta cell function are at lower risk of hypoglycaemia than those who have completely lost beta cell function [75]. The studies analysed in this review included relatively few participants with very short or very long duration of diabetes and also the age range was rather narrow, which limited our ability to detect an effect of diabetes duration or age. Second, it is also possible that age and/or diabetes duration affect the magnitude of counterregulatory responses to hypoglycaemia, rather than the glycaemic thresholds.

A strength of this systematic review is that the large number of participants provided more precision to estimate glycaemic thresholds for counterregulatory responses to hypoglycaemia with sufficient accuracy in both people with type 1 diabetes and people without diabetes. There are also limitations. First, the calculation of glycaemic thresholds depends on the level and number of glycaemic plateaus, the difference between the separate plateaus, and the number of participants, none of which were consistent across studies. However, we applied interval-censored data statistical methodology to control for these issues and two studies using a graded stepped hypoglycaemic clamp reported similar results [18, 19]. Additionally, three-step and four-step clamps resulted in broadly similar threshold levels and we found no evidence for a modulating effect of the number of participants in each study. Second, the relatively high doses of insulin used in most clamp studies may affect the counterregulatory hormone and symptom responses to hypoglycaemia, which could alter glycaemic thresholds when compared with hypoglycaemia occurring spontaneously. However, since such an effect would equally affect participants, this would not explain differences (or its absence) between participant subgroups or counterregulatory responses. Finally, we corrected for glucose levels measured in whole blood, assuming these to be 11% lower than glucose levels measured in plasma [70]. Although this relationship may be different under conditions of hyperinsulinaemia and hypoglycaemia because of different haematocrit levels [76], we considered this effect to be minimal.

The ADA defines hypoglycaemia in people with diabetes non-numerically as ‘all episodes of an abnormally low plasma glucose concentration that expose the individual to potential harm’ [77]. What constitutes a hypoglycaemic event in the treatment of diabetes is under debate and depends both on the setting and on individual factors. Indeed, it should be acknowledged that glucose levels below which counterregulatory responses are elicited show high intra- and interindividual variability, and this is supported by the ranges around the median glycaemic threshold levels reported here, particularly in people with diabetes. Nevertheless, our analysis in people without diabetes are in line with the International Hypoglycaemia Study Group (IHSG) classification for hypoglycaemia, particularly regarding level 1 hypoglycaemia [78]. However, it should be appreciated that this analysis was based on data obtained during experimental hypoglycaemia and that evidence from other sources, particularly spontaneous hypoglycaemia in daily clinical practice, is additionally needed to further solidify the IHSG classification.

In conclusion, this systematic review shows that counterregulatory hormone responses measured during stepped hyperinsulinaemic–hypoglycaemic glucose clamps are initiated at a median plasma glucose level of 3.8 mmol/l and that both autonomic and neuroglycopenic hypoglycaemic symptoms start at similar glucose levels of around 3.4 mmol/l in people without diabetes. In people with type 1 diabetes, the release of counterregulatory hormones and generation of warning symptoms occur at glucose levels that are 0.1–0.4 mmol/l lower than in people without diabetes, and sometimes at even lower levels in those with impaired awareness. These data may inform clinical practice as well as the conduction of future clinical trials and studies investigating hypoglycaemia, and contribute to discussions about refining the classification of hypoglycaemia.

## Supplementary Information

ESM 1 (PDF 851 kb)

## Data Availability

The datasets generated during and/or analysed during the current study are available from the corresponding author on reasonable request.

## Abbreviations

IHSG: International Hypoglycaemia Study Group
N/A: Not applicable/calculable
