# Supplementary material for: Glycaemic thresholds for counterregulatory hormone and symptom responses to hypoglycaemia in people with and without type 1 diabetes: a systematic review
Source: Diabetologia. 2022 Jul 22;65(10):1601–12. doi: 10.1007/s00125-022-05749-8 (PMC9477942; doi:10.1007/s00125-022-05749-8)

ESM Methods

Search strategy for PubMed

(((((((((("hypoglycemic clamp" OR "hypoglycaemic clamp*" NOT (("hypoglycemic clamp*" OR "hypoglycaemic clamp*") AND Animals[Mesh:noexp]))) OR (("hyperinsulinemic clamp*") NOT ("hyperinsulinemic clamp*" AND Animals[Mesh:noexp]))) OR ((("glucose clamp technique"[MeSH Terms] OR "glucose clamp technique"[All Fields] OR "glucose clamp"[All Fields] OR "glucose clamps"[All Fields] OR "glucose clamping"[All Fields])) NOT (("glucose clamp technique"[MeSH Terms] OR "glucose clamp technique"[All Fields] OR "glucose clamp"[All Fields] OR "glucose clamps"[All Fields] OR "glucose clamping"[All Fields]) AND Animals[Mesh:noexp]))))))))) AND ((((((hypoglycemia OR hypoglycaemia)) NOT (((hypoglycemia) OR hypoglycaemia) AND Animals[Mesh:noexp]))) OR ((hypoglycemic OR hypoglycaemic)) NOT ((hypoglycemic OR hypoglycaemic) AND Animals[Mesh:noexp])))*

ESM Table 1: Characteristics of the 63 articles included in this systematic review

**Article**	**Year**	**Objective**	**Population**	**Glycaemic steps, *n***	**Outcomes**
Amiel et al.<br>Effect of intensive insulin therapy on glycemic thresholds for counterregulatory hormone release.<br>Diabetes Jul 1988;37(7):901-7	1988	To investigate the influence of improved glycemic control of diabetes on plasma glucose thresholds for initiating counterregulatory hormone release.	People without diabetes (*n*=7)	6	Adrenaline, noradrenaline, cortisol, growth hormone
Amiel SA et al.<br>Effect of antecedent glucose control on cerebral function during hypoglycemia.<br>Diabetes care Feb 1991;14(2):109-18	1991	To investigate cerebral adaptation to low blood glucose level	People with type 1 diabetes (*n*=9), People without diabetes (*n*=8)	4	Adrenaline, noradrenaline, cortisol, growth hormone
Bingham E et al.<br>The effects of KATP channel modulators on counterregulatory responses and cognitive function during acute controlled hypoglycaemia in healthy men: a pilot study.<br>Diabetic medicine: a journal of the British Diabetic Association Mar 2003;20(3):231-7	2003	To study the effects of agents that alter potassium adenosine triphosphate (KATP) channel activity in β-cells on cognitive function and counterregulatory hormone responses during acute hypoglycaemia	People without diabetes (*n*=10)	4	Adrenaline, noradrenaline, glucagon, cortisol, growth hormone, neuroglycopenic symptoms, autonomic symptoms
Boyle PJ et al.<br>Plasma glucose concentrations at the onset of hypoglycemic symptoms in patients with poorly controlled diabetes and in nondiabetics.<br>The New England journal of medicine Jun 1988;318(23):1487-92	1988	To study if symptoms of hypoglycemia occur at higher glucoseconcentra tions in patients with po orly controlled insulin-dependent diabetes mell itus than in persons without diabetes	People with diabetes (*n*=8)	4	Noradrenaline, cortisol, growth hormone
Boyle PJ et al.<br>Adaptation in brain glucose uptake following recurrent hypoglycemia.<br>Proceedings of the National Academy of Sciences of the United States of America Sep 1994;91(20):9352-6	1994	To investigate if adaptations occur to allow maintenance of normal rates of brain glucose uptake following recurrent hypoglycemia in man	People without diabetes (*n*=12)	4	Adrenaline
Brierley EJ et al.<br>Reduced awareness of hypoglycaemia in the elderly despite an intact counter-regulatory response.	1995	To study awareness of hypoglycaemia and its counterregulatory hormones	People without type 1 diabetes (*n*=13)	4	Adrenaline, glucagon, neuroglycopenic symptoms, autonomic symptoms

QJM : monthly journal of the Association of Physicians Jun 1995;88(6):439-45					
Carey M et al.<br>Opioid Receptor Activation Impairs Hypoglycemic Counterregulation in Humans.<br>Diabetes 11 2017;66(11):2764-2773	2017	To determine whether and how opioid receptor activation induces HAAF in humans	People without diabetes (*n*=12)	3	Growth hormone
Cranston I et al.<br>Restoration of hypoglycaemia awareness in patients with long-duration insulin-dependent diabetes.<br>Lancet (London, England) Jul 1994;344(8918):283-7	1994	To investigate the possibility of restoring awareness; symptomatic, cognitive, and hormonal responses to controlled hypoglycaemia were studied in insulin-dependent diabetic patients with long disease duration	People with type 1 diabetes (*n*=12)	6	Adrenaline, noradrenaline, growth hormone
Dagogo-Jack SE et al.<br>Hypoglycemia-associated autonomic failure in insulin-dependent diabetes mellitus. Recent antecedent hypoglycemia reduces autonomic responses to, symptoms of, and defense against subsequent hypoglycemia.<br>The Journal of clinical investigation Mar 1993;91(3):819-28	1993	To study if patients with insulin-dependent diabetes mellitus (IDDM), recent antecedent iatrogenic hypoglycemia is a major cause of hypoglycemia-associated autonomic failure	People with type 1 diabetes (*n*=26), People without diabetes (*n*=12)	5	Neuroglycopenic symptoms, autonomic symptoms
Dagogo-Jack S et al.<br>Physiological responses during hypoglycaemia induced by regular human insulin or a novel human analogue, insulin glargine.<br>Diabetes, obesity & metabolism Dec 2000;2(6):373-83	2000	To determine and compare insulin glargine and regular insulin in relation to hypoglycaemic symptoms	People with type 1 diabetes (*n*=13), People without diabetes (*n*=6)	4	Adrenaline, noradrenaline, glucagon, growth hormone
Dantz D et al.<br>Vascular endothelial growth factor: a novel endocrine defensive response to hypoglycemia.<br>The Journal of clinical endocrinology and metabolism Feb 2002;87(2):835-40	2002	To investigate if vascular endothelial growth factor (VEGF), is a potent regulator of blood vessel function, is a candidate hormone for facilitating glucose passage across the blood-brain barrier under critical conditions	People without diabetes (*n*=16)	4	Adrenaline, noradrenaline, glucagon, cortisol, growth hormone
de Galan BE et al.<br>Theophylline improves hypoglycemia unawareness in type 1 diabetes.<br>Diabetes Mar 2002;51(3):790-6	2002	To test the effect of theophylline on responses to hypoglycemia	People with type 1 diabetes (*n*=15), people	2	Adrenaline, noradrenaline, growth hormone

			without diabetes (*n*=15)		
de Galan BE et al.<br>Effect of 2 weeks of theophylline on glucose counterregulation in patients with type 1 diabetes and unawareness of hypoglycemia.<br>Clinical pharmacology and therapeutics Jul 2003;74(1):77-84	2003	To assess the effect of theophylline oral 3 weeks prior to clamp	People with type 1 diabetes (*n*=12)	2	Adrenaline, noradrenaline, glucagon, growth hormone
de Galan BE et al.<br>Hypoglycaemia downregulates endotoxin-induced production of tumour necrosis factor-alpha, but does not affect IL-1beta, IL-6, or IL-10.<br>Cytokine May 2003;22(3-4):71-6	2003	To investigate the effect of hypoglycaemia on the production of the proinflammatory cytokines tumour necrosis factor-a (TNFa) and interleukin-1b (IL-1b) in subjects with and without diabetes	People with type 1 diabetes (*n*=9), people without diabetes (*n*=9)	2	Adrenaline, noradrenaline, cortisol
Deininger E et al.<br>Losartan attenuates symptomatic and hormonal responses to hypoglycemia in humans.<br>Clinical pharmacology and therapeutics Oct 2001;70(4):362-9	2001	Investigate the effects of losartan on symptomatic and hormonal responses to hypoglycaemia in humans	People without diabetes (*n*=16)	4	Adrenaline, noradrenaline, cortisol
Evans ML et al.<br>Reduced counterregulation during hypoglycemia with raised circulating nonglucose lipid substrates: evidence for regional differences in metabolic capacity in the human brain?<br>The Journal of clinical endocrinology and metabolism Aug 1998;83(8):2952-9	1998	To assess the potential for the human brain to use lipid fuels during acute hypoglycemia	People without diabetes (*n*=10)	4	Adrenaline, noradrenaline, glucagon, cortisol, growth hormone, neuroglycopenic symptoms, autonomic symptoms
Fanelli C et al.<br>Long-term recovery from unawareness, deficient counter regulation and lack of cognitive dysfunction during hypoglycaemia, following institution of rational, intensive insulin therapy in IDDM.<br>Diabetologia Dec 1994;37(12):1265-76	1994	To test if hypoglycaemia unawareness is reversible as long as hypoglycaemia is meticulously prevented and that intensive insulin therapy can maintain long-term near normoglycaemia.	People with type 1 diabetes (*n*=21), People without diabetes (*n*=20)	4	Adrenaline, noradrenaline, glucagon, cortisol, growth hormone, neuroglycopenic symptoms, autonomic symptoms
Fanelli C et al.<br>Effects of recent, short-term hyperglycemia on responses to hypoglycemia in humans.<br>Relevance to the pathogenesis of hypoglycemia unawareness and	1995	To assess whether short-term, antecedent hyperglycemia exerts effects opposite to those observed after acute hypoglycemia	People with type 1 diabetes (*n*=8), People without diabetes (*n*=7)	4	Adrenaline, noradrenaline, glucagon, cortisol, growth hormone, neuroglycopenic symptoms, autonomic symptoms

hyperglycemia-induced insulin resistance.<br>Diabetes May 1995;44(5):513-9					
Fanelli CG et al.<br>Impact of nocturnal hypoglycemia on hypoglycemic cognitive dysfunction in type 1 diabetes.<br>Diabetes Dec 1998;47(12):1920-7	1998	The impact of nocturnal hypoglycaemia in cognitive function	People with type 1 diabetes (*n*=15), People without diabetes (*n*=12)	4	Adrenaline, noradrenaline, neuroglycopenic symptoms, autonomic symptoms
Fanelli C et al.<br>Long-term intensive therapy of IDDM patients with clinically overt autonomic neuropathy: effects on hypoglycemia awareness and counterregulation.<br>Diabetes Jul 1997;46(7):1172-81	1997	To test if hypoglycemia unawareness and impaired counterregulation are reversible	People with type 1 diabetes (*n*=21), People without diabetes (*n*=15)	4	Adrenaline, noradrenaline, glucagon, cortisol, growth hormone, neuroglycopenic symptoms, autonomic symptoms
Fanelli CG et al.<br>Meticulous prevention of hypoglycemia normalizes the glycemic thresholds and magnitude of most of neuroendocrine responses to, symptoms of, and cognitive function during hypoglycemia in intensively treated patients with short-term IDDM.<br>Diabetes Nov 1993;42(11):1683-9	1993	To test the hypothesis that hypoglycemia unawareness is largely secondary to recurrent therapeutic hypoglycemia in IDDM	People with type 1 diabetes (*n*=8), People without diabetes (*n*=12)	4	Adrenaline, glucagon, neuroglycopenic symptoms, autonomic symptoms
Fanelli C et al.<br>Relative roles of insulin and hypoglycaemia on induction of neuroendocrine responses to, symptoms of, and deterioration of cognitive function in hypoglycaemia in male and female humans.<br>Diabetologia Aug 1994;37(8):797-807	1994	To assess the relative roles of insulin and hypoglycaemia on induction of neuroendocrine responses, symptoms and deterioration of cognitive function	People without diabetes (*n*=22)	4	Adrenaline, noradrenaline, glucagon, cortisol, growth hormone, neuroglycopenic symptoms, autonomic symptoms
Fruehwald-Schultes B et al.<br>Adaptation of cognitive function to hypoglycemia in healthy men.<br>Diabetes care Aug 2000;23(8):1059-66	2000	To test the effect of antecedent hypoglycemia on hypoglycemic counterregulation and symptoms	People with type 1 diabetes (*n*=30)	4	Adrenaline, cortisol, growth hormone
Gabriely I et al.<br>Fructose normalizes specific counterregulatory responses to hypoglycemia in patients with type 1 diabetes.<br>Diabetes Mar 2005;54(3):609-16	2005	The effect of fructose to counterregulatory hormone responses to hypoglycaemia	People with type 1 diabetes (*n*=8)	3	Growth hormone
Gabriely I et al.	2002	Examined the effects of an acute infusion of	People without	3	Adrenaline, growth hormone

Fructose amplifies counterregulatory responses to hypoglycemia in humans.Diabetes Apr 2002;51(4):893-900		fructose on hypoglycemia counterregulation	diabetes (*n*=7)		
Gabriely I et al. Troglitazone amplifies counterregulatory responses to hypoglycemia in nondiabetic subjects.The Journal of clinical endocrinology and metabolism Feb 2001;86(2):521-8	2001	Evaluated the effects of a short course of troglitazone on counterregulatory hormones in response to mild hypoglycemia	People without diabetes (*n*=8)	3	Noradrenaline, glucagon, cortisol, growth hormone
George E et al. Preservation of physiological responses to hypoglycemia 2 days after antecedent hypoglycemia in patients with IDDM. Diabetes care Aug 1997;20(8):1293-8	1997	To assess the effects of short-term antecedent hypoglycemia on responses to further hypoglycemia 2 days later in patients with IDDM	People with type 1 diabetes (*n*=8)	3	Adrenaline, neuroglycopenic symptoms, autonomic symptoms
Hermanns N et al. Effect of experimentally induced hypoglycemia and different insulin levels on feelings of hunger in type 1 diabetic patients. Experimental and clinical endocrinology & diabetes: official journal, German Society of Endocrinology [and] German Diabetes Association May 2008;116(5):255-61	2008	Investigate the impacts of experimentally induced hypoglycemia and different insulin infusion rates on feelings of hunger	People with type 1 diabetes (*n*=16)	3	Adrenaline, growth hormone, neuroglycopenic symptoms, autonomic symptoms
Jones TW et al. Resistance to neuroglycopenia: an adaptive response during intensive insulin treatment of diabetes. The Journal of clinical endocrinology and metabolism Jun 1997;82(6):1713-8	1997	To determine whether counterregulation and awareness of hypoglycaemia are associated with an alteration in the susceptibility of the brain to mild hypoglycemia	People with type 1 diabetes (*n*=19), People without diabetes (*n*=10)	4	Adrenaline, noradrenaline, cortisol, growth hormone
Jones TW et al. Mild hypoglycemia and impairment of brain stem and cortical evoked potentials in healthy subjects. Diabetes Dec 1990;39(12):1550-5	1990	To evaluate the impact of mild hypoglycemia on CNS function in healthy adults	People without diabetes (*n*=14)	3	Adrenaline, growth hormone
Jones TW et al. Independent effects of youth and poor diabetes control on responses to hypoglycemia in children. Diabetes Mar 1991;40(3):358-63	1991	To evaluate the effects of childhood and poorly controlled insulin-dependent diabetes mellitus	People with type 1 diabetes (*n*=13), People without	5	Glucagon, noradrenaline, cortisol, growth hormone

			diabetes (*n*=32)		
Kanc K et al.<br>Substitution of night-time continuous subcutaneous insulin infusion therapy for bedtime NPH insulin in a multiple injection regimen improves counterregulatory hormonal responses and warning symptoms of hypoglycaemia in IDDM.<br>Diabetologia Mar 1998;41(3):322-9	1998	Assessed the influence of a more stable nocturnal blood glucose control on the counterregulatory hormonal responses and symptoms of hypoglycaemia.	People with type 1 diabetes (*n*=14), People without diabetes (*n*=12)	5	Adrenaline, noradrenaline, cortisol, growth hormone, neuroglycopenic symptoms
Kerr D et al.<br>Adaptation to mild hypoglycaemia in normal subjects despite sustained increases in counter-regulatory hormones.<br>Diabetologia Apr 1989;32(4):249-54	1989	The effects of adaptation to mild hypoglycaemia	People without diabetes (*n*=7)	3	Adrenaline, cortisol, growth hormone
Kerr D et al.<br>Influence of duration of hypoglycemia on the hormonal counterregulatory response in normal subjects.<br>The Journal of clinical endocrinology and metabolism Jun 1989;68(6):1118-22	1989	To investigate the counterregulatory response to recurrent and prolonged mild hypoglycemia in women without diabetes	People without diabetes (*n*=6)	2	Adrenaline, noradrenaline, cortisol, growth hormone
Kinsley BT et al.<br>Stimulus specificity of defects in counterregulatory hormone secretion in insulin-dependent diabetes mellitus: effect of glycemic control.<br>The Journal of clinical endocrinology and metabolism Nov 1994;79(5):1383-9	1994	The effect of counterregulatory hormone responses to hypoglycemia in subjects with insulin-dependent diabetes mellitus (IDDM) in strict glycemic control	People with type 1 diabetes (*n*=18), People without diabetes (*n*=10)	5	Noradrenaline, cortisol
Kinsley BT et al.<br>Differential regulation of counterregulatory hormone secretion and symptoms during hypoglycemia in IDDM. Effect of glycemic control.<br>Diabetes care Jan 1995;18(1):17-26	1995	To investigate if there was a differential effect of glycemic control on individual counterregulatory hormone responses to hypoglycemia in patients	People with type 1 diabetes (*n*=38), People without diabetes (*n*=38)	5	Adrenaline, noradrenaline, cortisol, growth hormone
Kinsley BT et al.<br>Evidence for a hypothalamic-pituitary versus adrenal cortical effect of glycemic control on counterregulatory hormone responses to hypoglycemia in insulin-dependent diabetes mellitus.	1996	To determine the effect of hypothalamic-pituitary versus adrenal cortical effect of glycemic control on counterregulatory hormone responses to hypoglycemia	People with type 1 diabetes (*n*=27), People without diabetes (*n*=20)	5	Adrenaline, cortisol, growth hormone

The Journal of clinical endocrinology and metabolism Feb 1996;81(2):684-91					
Koivikko ML et al. Effects of sustained insulin-induced hypoglycemia on cardiovascular autonomic regulation in type 1 diabetes.<br>Diabetes Mar 2005;54(3):744-50	2005	Effects of hypoglycemia on cardiac autonomic regulation	People with type 1 diabetes (*n*=16), People without diabetes (*n*=8)	3	Adrenaline, noradrenaline, glucagon, cortisol
Leelarathna L et al.<br>Restoration of self-awareness of hypoglycemia in adults with long-standing type 1 diabetes: hyperinsulinemic-hypoglycemic clamp substudy results from the HypoCOMPaSS trial.<br>Diabetes care Dec 2013;36(12):4063-70	2013	To demonstrate that by optimizing conventional management, including the use of real time continuous glucose monitoring in individuals with DM1 complicated by IAH, rigorous prevention of BH will restore awareness and reduce risk of recurrent SH	People with type 1 diabetes (*n*=18)	4	Growth hormone, neuroglycopenic symptoms, autonomic symptoms
Maran A et al.<br>Lack of preservation of higher brain function during hypoglycaemia in patients with intensively treated IDDM.<br>Diabetologia Dec 1995;38(12):1412-8,	1995	To investigate the effect of diabetes control on higher brain function during acute hypoglycaemia	People with type 1 diabetes (*n*=16), People without diabetes (*n*=8)	3	Adrenaline
Maran A et al.<br>Brain function rescue effect of lactate following hypoglycaemia is not an adaptation process in both normal and type I diabetic subjects.<br>Diabetologia Jun 2000;43(6):733-41	2000	To test if there is an lactate adaption during hypoglycaemia	People with type 1 diabetes (*n*=7), People without diabetes (*n*=7)	2	Adrenaline, noradrenaline, cortisol, growth hormone, neuroglycopenic symptoms, autonomic symptoms
Maran A et al.<br>Cognitive, neurophysiologic and metabolic sequelae of previous hypoglycemic coma revealed by hyperinsulinemic-hypoglycemic clamp in type 1 diabetic patients.<br>Metabolic brain disease 10 2017;32(5):1543-1551	2017	To examine the relationship between electroencephalographic (EEG) activity and hypoglycemia unawareness	People with type 1 diabetes (*n*=12)	3	Adrenaline, growth hormone
Matyka K et al.<br>Altered hierarchy of protective responses against severe hypoglycemia in normal aging in healthy men.<br>Diabetes care Feb 1997;20(2):135-41	1997	To investigate the effect of normal aging on the protective responses against hypoglycemia	People without diabetes (*n*=14)	4	Adrenaline, noradrenaline, glucagon, cortisol, growth hormone, neuroglycopenic symptoms, autonomic symptoms

Mellman MJ et al.<br>Effect of antecedent hypoglycemia on cognitive function and on glycemic thresholds for counterregulatory hormone secretion in healthy humans.<br>Diabetes care Mar 1994;17(3):183-8	1994	To determine whether reduced hormonal, symptomatic, and/or cognitive responses to hypoglycemia are caused by an increase in the plasma glucose concentration	People without diabetes (*n*=9)	3	Adrenaline, noradrenaline, glucagon, cortisol, growth hormone
Meneilly G et al.<br>Counterregulatory hormone responses to hypoglycemia in the elderly patient with diabetes.<br>Diabetes Mar 1994;43(3):403-10	1994	To investigate counterregulatory hormone responses to hypoglycemia in elderly patients	People with type 1 diabetes (*n*=10), People without diabetes (*n*=10)	4	Adrenaline, noradrenaline, glucagon, cortisol, growth hormone, neuroglycopenic symptoms, autonomic symptoms
Meneilly GS et al.<br>Altered responses to hypoglycemia of healthy elderly people.<br>The Journal of clinical endocrinology and metabolism Jun 1994;78(6):1341-8	1994	To assess whether alterations in counterregulatory hormone release, decreased awareness of warning symptoms or alterations in psychomotor performance might increase the susceptibility of the elderly to hypoglycemia	People without diabetes (*n*=19)	4	Adrenaline, noradrenaline, glucagon, cortisol, growth hormone
Meyer C et al.<br>Improved glucose counterregulation and autonomic symptoms after intraportal islet transplants alone in patients with long-standing type I diabetes mellitus.<br>Transplantation Jul 1998;66(2):233-40	1998	To asses if successful intraportal islet transplantation affect hormonal counterregulatory responses and symptoms	People with type 1 diabetes (*n*=3), People without diabetes (*n*=10)	4	Adrenaline, noradrenaline, cortisol, growth hormone, neuroglycopenic symptoms, autonomic symptoms
Meyer C, et al.<br>Effects of autonomic neuropathy on counterregulation and awareness of hypoglycemia in type 1 diabetic patients.<br>Diabetes care Nov 1998;21(11):1960-6	1998	The effect of autonomic neuropathy on counterregulation and awareness of hypoglycemia	People without diabetes (*n*=19)	4	Adrenaline, noradrenaline, glucagon, cortisol, growth hormone, neuroglycopenic symptoms, autonomic symptoms
Mitrakou A et al.<br>Hierarchy of glycemic thresholds for counterregulatory hormone secretion, symptoms, and cerebral dysfunction.<br>The American journal of physiology Jan 1991;260(1 Pt 1):E67-74	1991	To investigate hierarchy of glycemic thresholds for counterregulatory hormone secretion	People without diabetes (*n*=10)	4	Adrenaline, noradrenaline, glucagon, cortisol, growth hormone, neuroglycopenic symptoms, autonomic symptoms

Mokan M et al.<br>Hypoglycemia unawareness in IDDM.<br>Diabetes care Dec 1994;17(12):1397-403	1994	To assess the characteristics of patients with hypoglycemia unawareness	People with type 1 diabetes (*n*=43), People without diabetes (*n*=19)	4	Adrenaline, noradrenaline, cortisol, growth hormone, neuroglycopenic symptoms, autonomic symptoms
Oltmanns KM et al.<br>Influence of captopril on symptomatic and hormonal responses to hypoglycaemia in humans.<br>British journal of clinical pharmacology Apr 2003;55(4):347-53	2003	The effect of captopril on hypoglycaemia	People without diabetes (*n*=16)	3	Adrenaline, cortisol
Ovalle F et al.<br>Brief twice-weekly episodes of hypoglycemia reduce detection of clinical hypoglycemia in type 1 diabetes mellitus.<br>Diabetes Sep 1998;47(9):1472-9	1998	To assess the impact of hypoglycemia-associated autonomic failure on the daily lives of people with DM1	People with type 1 diabetes (*n*=6), People without diabetes (*n*=12)	4	Noradrenaline, cortisol
Powers WJ, Boyle PJ, Hirsch IB, Cryer PE (1993)<br>Unaltered cerebral blood flow during hypoglycemic activation of the sympathochromaffin system in humans.<br>The American journal of physiology Oct 1993;265(4 Pt 2):R883-7	1993	To determine if increases in plasma epinephrine and norepinephrine caused by hypoglycemia are associated with increments in cerebral blood flow	People without diabetes (*n*=7)	2	Adrenaline, glucagon
Rickels MR et al.<br>Glycemic thresholds for activation of counterregulatory hormone and symptom responses in islet transplant recipients.<br>The Journal of clinical endocrinology and metabolism Mar 2007;92(3):873-9	2007	To determine whether the avoidance of hypoglycemia achieve d through islet transplantation results in improved glycemic thresholds for counterregulatory respo nses	People with type 1 diabetes (*n*=13), People without diabetes (*n*=8)	4	Adrenaline, noradrenaline, glucagon, cortisol, growth hormone, autonomic symptoms
Schultes B et al.<br>Processing of food stimuli is selectively enhanced during insulin-induced hypoglycemia in healthy men.<br>Psychoneuroendocrinology Jun 2005;30(5):496-504	2005	To explore the neuroendocrine mechanisms mediating the acute effects of sleep deprivation on blood glucose regulation under basal and hypoglycemic conditions	People without diabetes (*n*=15)	3	Adrenaline, noradrenaline, glucagon, cortisol, growth hormone
Schwartz NS et al.<br>Glycemic thresholds for activation of glucose counterregulatory systems are	1987	To define glycemic thresholds for activation of glucose counterregulatory systems and for	People without diabetes (*n*=10)	5	Adrenaline, noradrenaline, glucagon, cortisol, growth hormone

higher than the threshold for symptoms.<br>The Journal of clinical investigation Mar 1987;79(3):777-81		symptoms of hypoglycemia			
Sherwin RS et al.<br>Evaluation of hypoglycemic counterregulation using a modification of the Andres glucose clamp.<br>Experimental gerontology ;28(4-5):371-80	1993	To study counterregulatory mechanisms against hypoglycemia	People with type 1 diabetes (*n*=20), People without diabetes (*n*=70)	3	Adrenaline
Smith D et al.<br>The effect of modafinil on counter-regulatory and cognitive responses to hypoglycaemia.<br>Diabetologia Oct 2004;47(10):1704-11	2004	To assess if reducing release of the inhibitory neurotransmitter gamma-aminobutyric acid (GABA) with modafinil will enhance symptomatic and hormonal responses to hypoglycaemia	People without diabetes (*n*=9)	5	Adrenaline, noradrenaline, glucagon, cortisol, growth hormone, neuroglycopenic symptoms, autonomic symptoms
Snorgaard O et al. Glycaemic thresholds for hypoglycaemic symptoms, impairment of cognitive function, and release of counterregulatory hormones in subjects with functional hypoglycaemia.<br>Journal of internal medicine Apr 1991;229(4):343-50	1991	To investigate if subjects with functional hypoglycaemia may have inappropriately high glycaemic thresholds for the onset and perception of hypoglycaemia, as well as for the release of counterregulatory hormones	People without diabetes (*n*=9)	3	Adrenaline, noradrenaline, glucagon, cortisol, growth hormone
Taverna MJ, et al.<br>Insufficient adaptation of hypoglycaemic threshold for cognitive impairment in tightly controlled type 1 diabetes.<br>Diabetes & metabolism Feb 2000;26(1):58-64	2000	To investigate the threshold for cognitive impairment	People with type 1 diabetes (*n*=19)	5	Adrenaline, noradrenaline, glucagon, cortisol, growth hormone, neuroglycopenic symptoms, autonomic symptoms
ter Braak EW et al.<br>The sulfonylurea glyburide induces impairment of glucagon and growth hormone responses during mild insulin-induced hypoglycemia.<br>Diabetes care Jan 2002;25(1):107-12	2002	Investigating the impact of glyburide on glucose counterregulatory hormones during stepwise hypoglycemic clamp studies	People without diabetes (*n*=16)	3	Growth hormone
Vea H et al.<br>Reproducibility of glycaemic thresholds for activation of counterregulatory hormones and hypoglycaemic symptoms in healthy subjects.	1992	To look at the reproducibility of hypoglycaemic thresholds for activation of neuroendocrine	People without diabetes (*n*=9)	5	Adrenaline, glucagon, cortisol, growth hormone, neuroglycopenic symptoms, autonomic symptoms

Diabetologia Oct 1992;35(10):958-61		responses and symptoms			
Weinger K et al.<br>Blood glucose estimation and symptoms during hyperglycemia and hypoglycemia in patients with insulin-dependent diabetes mellitus.<br>The American journal of medicine Jan 1995;98(1):22-31	1995	Determine hypoglycemic and hyperglycemic symptoms, accuracy of estimating blood glucose, and their relation to glycemic control and counterregulatory hormone levels	People with type 1 diabetes (*n*=42)	3	Adrenaline, noradrenaline, cortisol

ESM Table 2 Associations between glycaemic thresholds for counterregulatory hormone or symptom responses and HbA_{1c} or diabetes duration.

	HbA_{1c} ***Hazard ratio*** ***(95% CI)***	***p-value***	Diabetes duration *Hazard ratio* *(95% CI)*	*p-value*
**Adrenaline**	1.081 (0.703-1.663)	0.723	1.007 (0.911-1.113)	0.893
**Noradrenaline**	0.896 (0.674-1.192)	0.452	0.990 (0.903-1.086)	0.832
**Cortisol**	0.797 (0.564-1.125)	0.197	0.977 (0.893-1.070)	0.618
**Growth Hormone**	0.762 (0.567-1.023)	0.071	1.023 (0.959-1.092)	0.485
**Autonomic symptoms**	0.735 (0.401-1.345)	0.317	1.143 (0.906-1.443)	0.259
**Neuroglycopenic symptoms**	1.192 (0.627-2.265)	0.593	1.033 (0.930-1.147)	0.542

Hazard ratios are shown per 1% HbA_{1c} increase and per 1 year longer diabetes duration. A hazard ratio < 1 means that if the HbA_{1c} or diabetes duration increases, the threshold is at higher glucose leve

ESM Fig.1 PRISMA-flow diagram

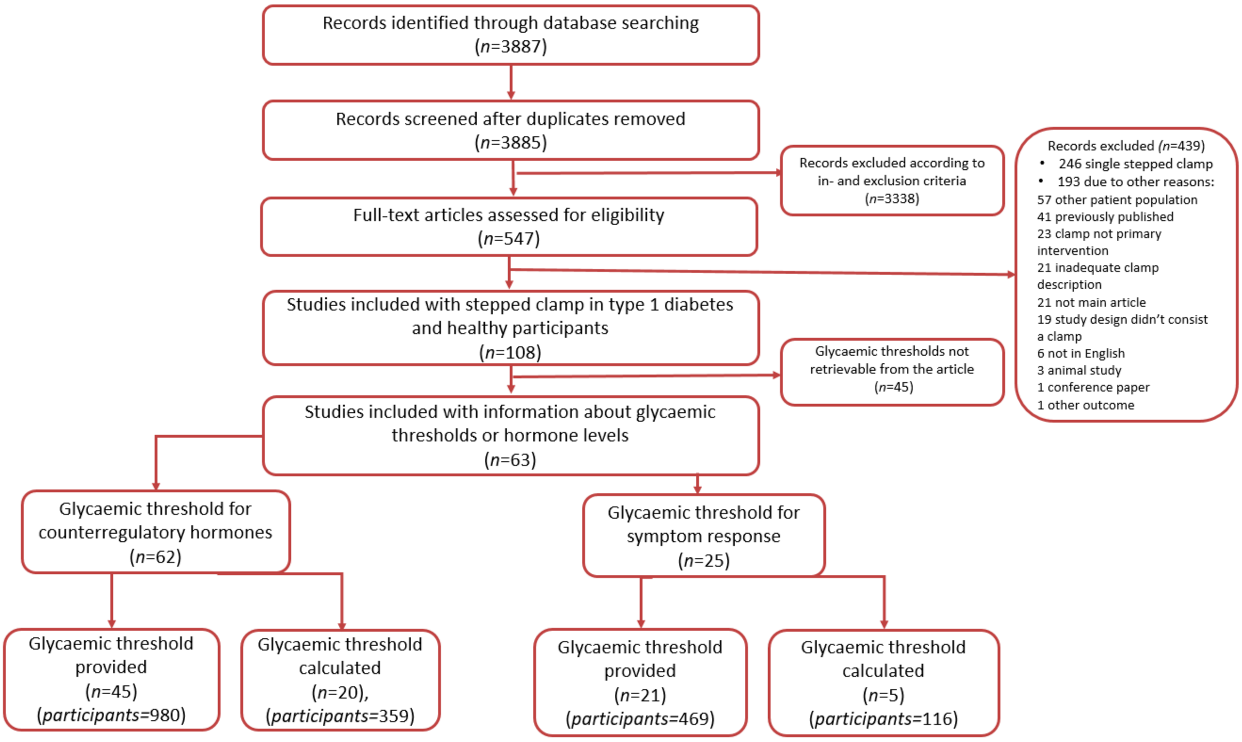

ESM Fig. 2 Glycaemic thresholds of counterregulatory and symptomatic responses to hypoglycaemia from the individual papers included both people with and without type 1 diabetes. The p-values refer to the comparison of curves of people with type 1 diabetes and without diabetes with a generalized log-rank test for interval censored survival curves. Data are shown in non-parametric complementary cumulative distribution curves (‘survival’ curves).

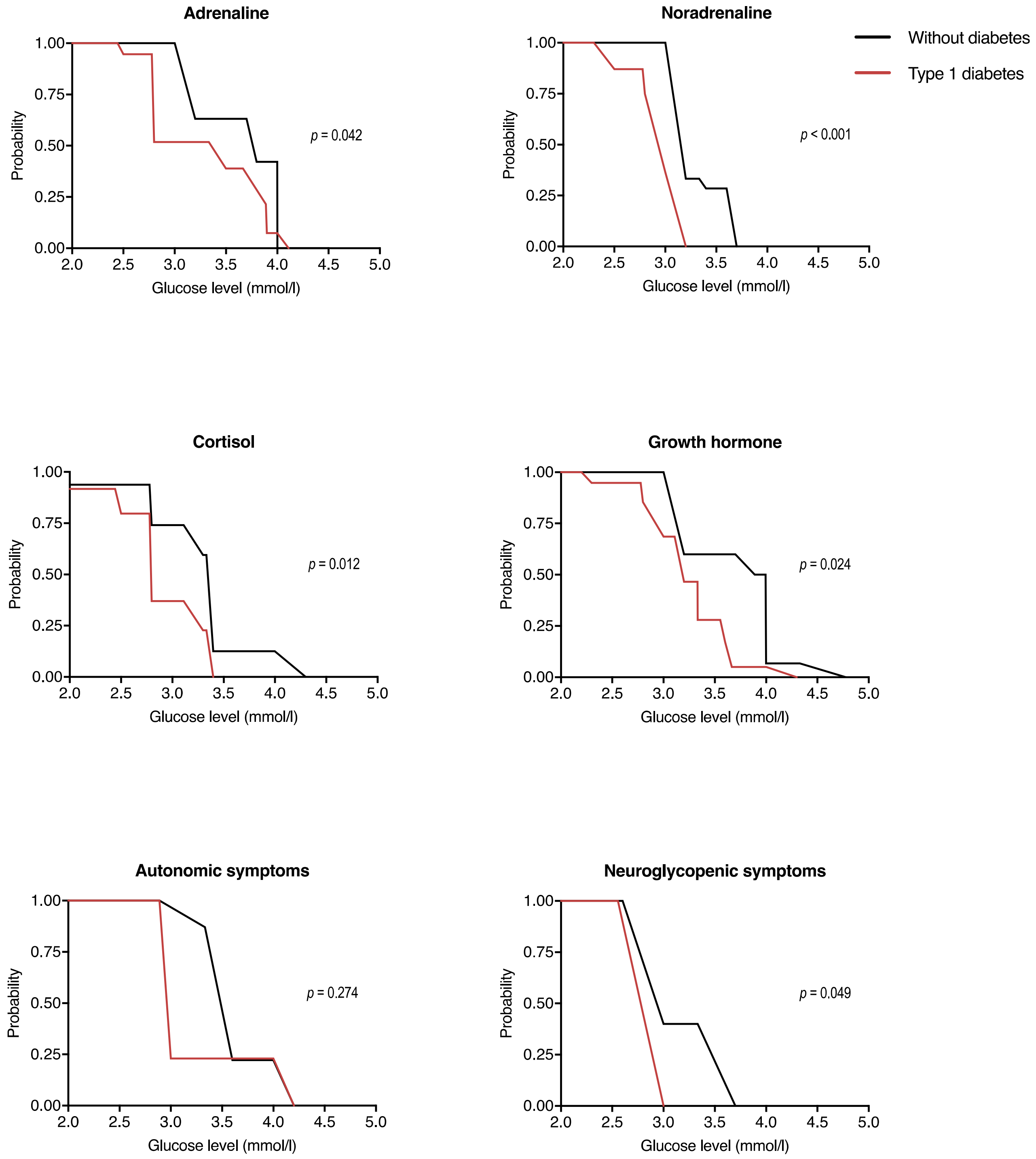

ESM. Fig. 3 Glycaemic thresholds for counterregulatory and symptomatic responses to hypoglycaemia from individual papers determined thresholds that either did or did not include a euglycaemic control clamp in people with type 1 diabetes (A) and people without diabetes (B). The p-values refer to the comparison of curves of clamps that included a euglycaemic control clamp or did not with a generalized log-rank test for interval censored survival curves. Data are shown in non-parametric complementary cumulative distribution curves ('survival' curves).

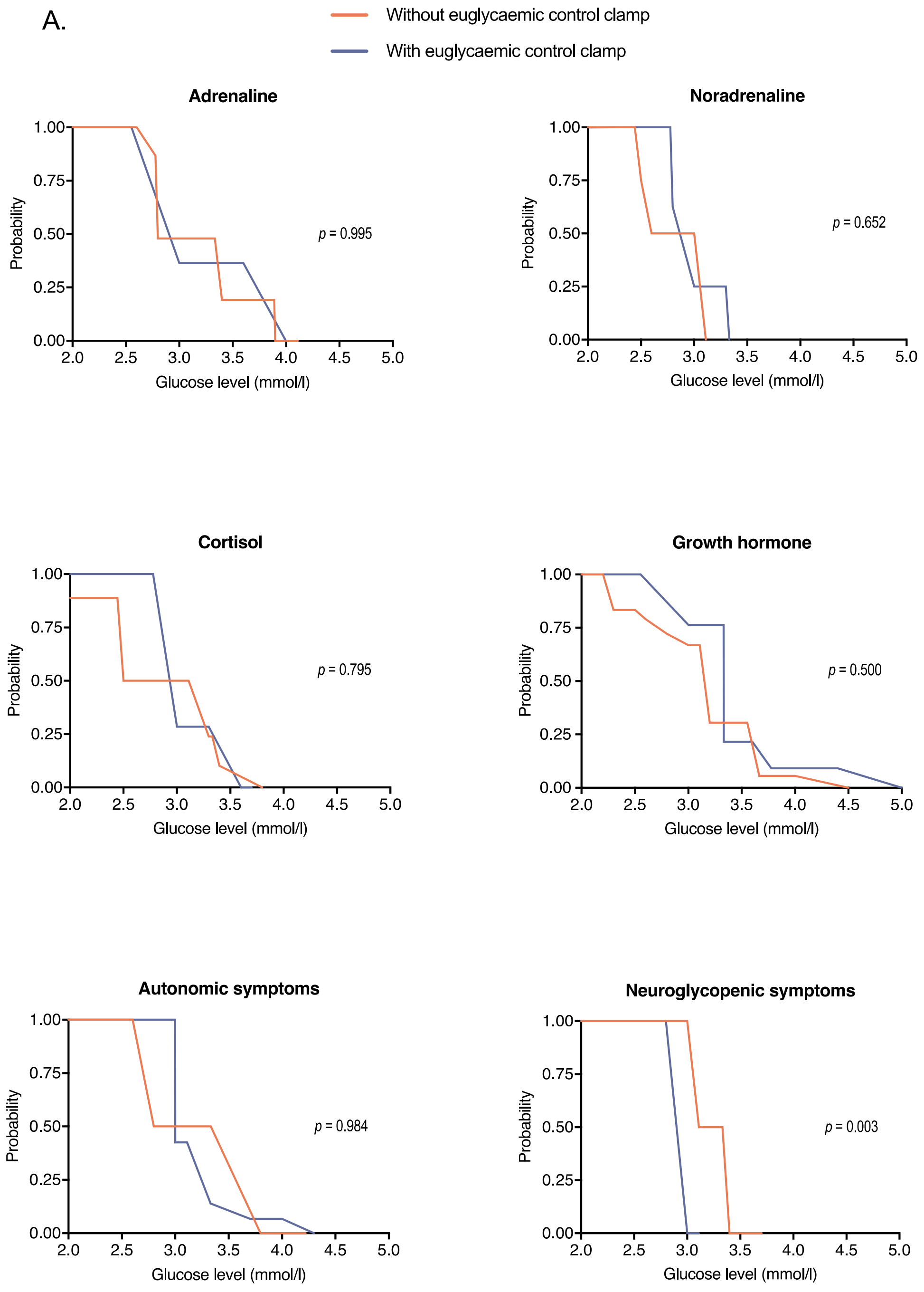

B.

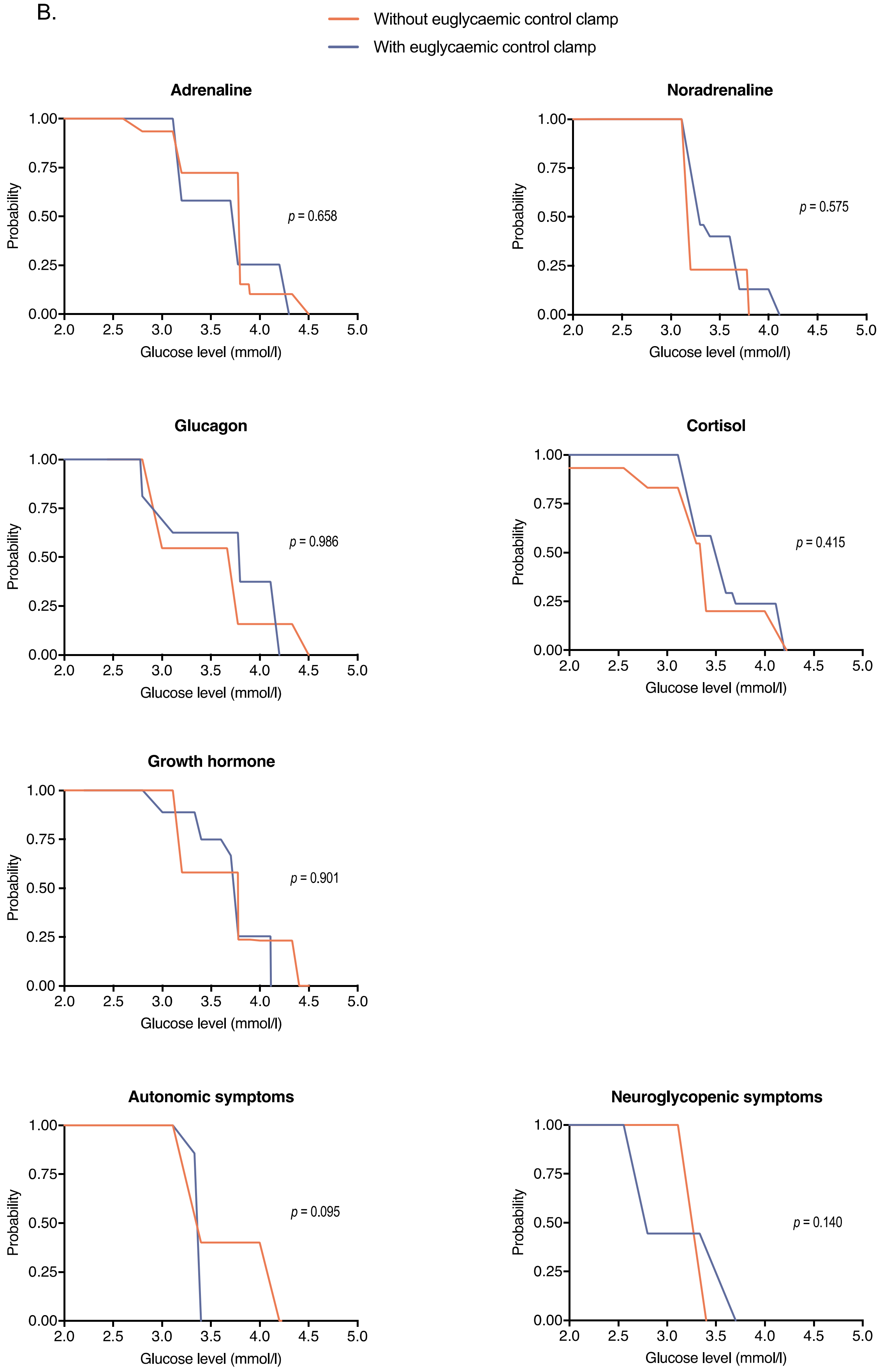

Without euglycaemic control clamp
With euglycaemic control clamp
Adrenaline
p = 0.658
Noradrenaline
p = 0.575
Glucagon
p = 0.986
Cortisol
p = 0.415
Growth hormone
p = 0.901
Autonomic symptoms
p = 0.095
Neuroglycopenic symptoms
p = 0.140
Probability
1.00
0.75
0.50
0.25
0.00
Glucose level (mmol/l)
2.0
2.5
3.0
3.5
4.0
4.5
5.0

ESM Fig. 4 Glycaemic thresholds of counterregulatory and symptomatic responses to hypoglycaemia from the individual papers included people with type 1 diabetes with either normal awareness of hypoglycaemia (NAH) or impaired awareness of hypoglycaemia (IAH). The p-values refer to the comparison of curves of NAH and IAH with a generalized log-rank test for interval censored survival curves. Data are shown in non-parametric complementary cumulative distribution curves (‘survival’ curves).

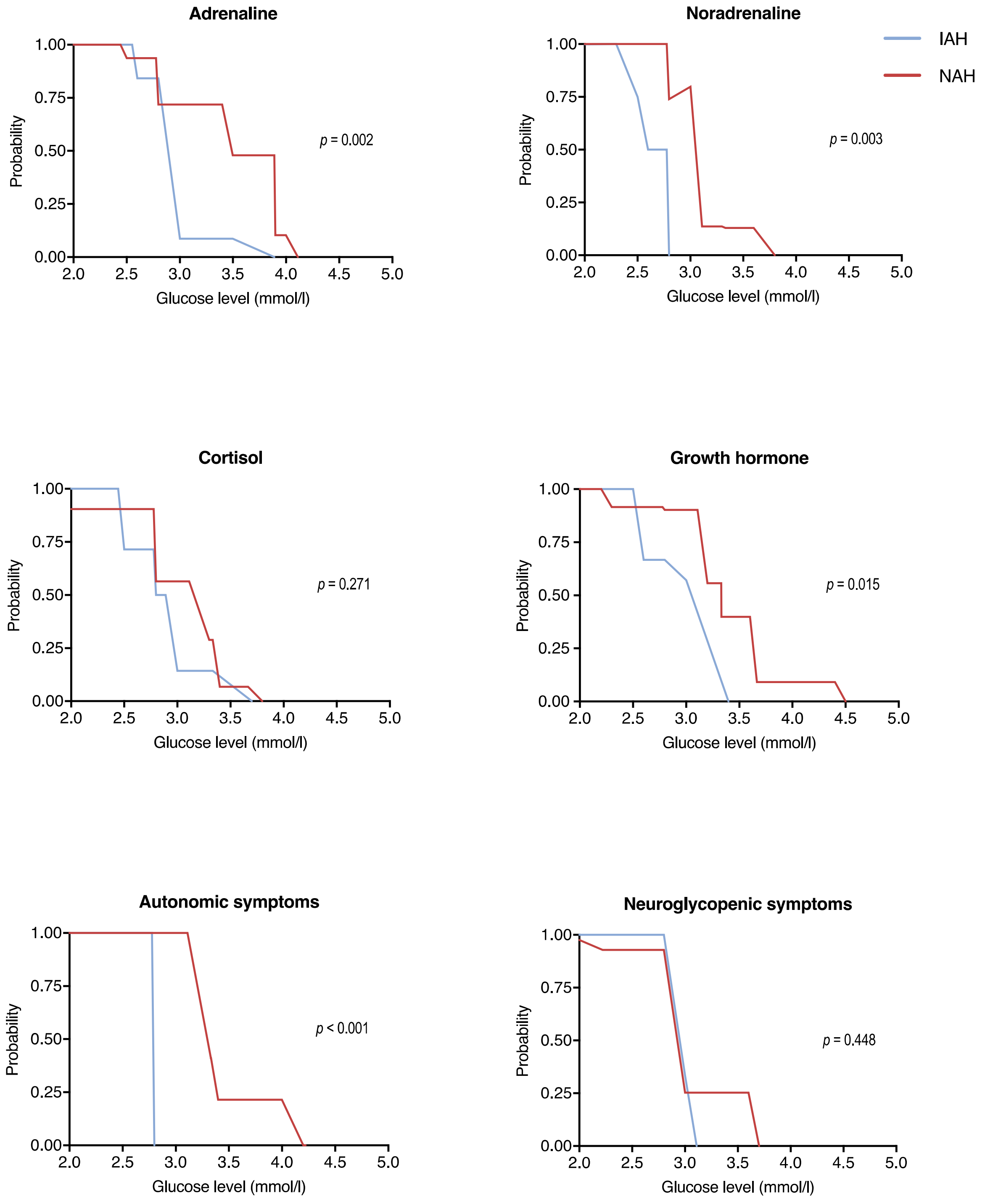

Supplement: Supplementary file 1 — (PDF 851 kb) [file 125_2022_5749_MOESM1_ESM.pdf]
